# Carbamazepine Potentiates the Effectiveness of Morphine in a Rodent Model of Neuropathic Pain

**DOI:** 10.1371/journal.pone.0107399

**Published:** 2014-09-15

**Authors:** Michael R. Due, Xiao-Fang Yang, Yohance M. Allette, Aaron L. Randolph, Matthew S. Ripsch, Sarah M. Wilson, Erik T. Dustrude, Rajesh Khanna, Fletcher A. White

**Affiliations:** 1 Department of Anesthesia, Indiana University School of Medicine, Indianapolis, Indiana, United States of America; 2 Department of Pharmacology, College of Medicine, University of Arizona, Tucson, Arizona, United States of America; 3 Department of Cell Biology and Anatomy, Indiana University School of Medicine, Indianapolis, Indiana, United States of America; 4 Program in Medical Neurosciences, Paul and Carole Stark Neurosciences Research Institute, Indiana University School of Medicine, Indianapolis, Indiana, United States of America; 5 Department of Pharmacology and Toxicology, Indiana University School of Medicine, Indianapolis, Indiana, United States of America; University of Sao Paulo, Brazil

## Abstract

Approximately 60% of morphine is glucuronidated to morphine-3-glucuronide (M3G) which may aggravate preexisting pain conditions. Accumulating evidence indicates that M3G signaling through neuronal Toll-like receptor 4 (TLR4) may be central to this proalgesic signaling event. These events are known to include elevated neuronal excitability, increased voltage-gated sodium (NaV) current, tactile allodynia and decreased opioid analgesic efficacy. Using an *in vitro* ratiometric-based calcium influx analysis of acutely dissociated small and medium-diameter neurons derived from lumbar dorsal root ganglion (DRG), we observed that M3G-sensitive neurons responded to lipopolysaccharide (LPS) and over 35% of these M3G/LPS-responsive cells exhibited sensitivity to capsaicin. In addition, M3G-exposed sensory neurons significantly increased excitatory activity and potentiated NaV current as measured by current and voltage clamp, when compared to baseline level measurements. The M3G-dependent excitability and potentiation of NaV current in these sensory neurons could be reversed by the addition of carbamazepine (CBZ), a known inhibitor of several NaV currents. We then compared the efficacy between CBZ and morphine as independent agents, to the combined treatment of both drugs simultaneously, in the tibial nerve injury (TNI) model of neuropathic pain. The potent anti-nociceptive effects of morphine (5 mg/kg, i.p.) were observed in TNI rodents at post-injury day (PID) 7–14 and absent at PID21–28, while administration of CBZ (10 mg/kg, i.p.) alone failed to produce anti-nociceptive effects at any time following TNI (PID 7–28). In contrast to either drug alone at PID28, the combination of morphine and CBZ completely attenuated tactile hyperalgesia in the rodent TNI model. The basis for the potentiation of morphine in combination with CBZ may be due to the effects of a latent upregulation of NaV1.7 in the DRG following TNI. Taken together, our observations demonstrate a potential therapeutic use of morphine and CBZ as a combinational treatment for neuropathic pain.

## Introduction

Morphine is the cornerstone of pain management in a number of pain conditions. However, therapeutic administration of morphine or morphine equivalents for neuropathic pain is limited to the second or third line of medication due to multiple adverse effects including incomplete efficacy [1], respiratory depression, and induction of sedation, constipation, nausea, vomiting, addiction and tolerance [2]. It has also been reported both experimentally and clinically that exposure to morphine can elicit a paradoxical pain in regions of the body unrelated to the initial pain complaint, aggravating preexisting pain; opioid-induced hyperalgesia (OIH) [3]. However, the molecular mechanisms of OIH are largely unknown. Numerous groups have suggested that OIH may be due to tonic, descending facilitation in the spinal cord [4], [5] or in the spinal cord dorsal horn [6], [7] through changes in activity of NMDA receptors. Though neither of these modes of action can be completely ruled out, an alternative OIH mechanism involves functional activation of the innate immune receptor Toll-like receptor 4 (TLR4) known to exhibited by a subpopulation of nociceptive neurons [8], [9].

TLR4 is a member of a fundamental family of receptors known for their ability to recognize pathogen-associated molecular patterns and facilitate the production of pro-inflammatory cytokines. TLR4 activation is not limited to pathogen detection; recent publications suggest that opioids and their glucuronide metabolites, among other ligands, can elicit TLR4-dependent activation in glial and neuronal cells [9], [10], [11], [12], [13], [14]. Moreover, both pharmacological blockade of TLR4 signaling and the use of TLR4 knockout mice serve to prevent behavioral hyperalgesia due to morphine tolerance and potentiate acute morphine analgesia *in vivo* [9], [15], [16].

Molecular modeling of opioid receptors has provided key information regarding the affinity of a variety of opioid ligands. Yet relatively little is known regarding the opioid-mediated TLR4 receptor action on neurons associated with the nociceptive pathway. A recent investigation using the morphine metabolite, morphine-3-glucuronide (M3G) to study TLR4-mediated activation in small diameter nociceptive neurons revealed that the there is a substantial increase in the current density for voltage-gated sodium channels (NaV), NaV1.6, NaV1.7 and NaV1.9, but not NaV1.8. [9]. The fact that these Na^+^ currents were influenced by a TLR4 agonist suggests that pharmacological targeting may provide mechanistic insights into both OIH and neuropathic disorders.

A suitable candidate to potentially diminish the neuronal TLR4 agonist-mediated signaling increases in excitation via sodium current modulation, is the anti-epileptic drug carbamazepine (CBZ) [17]. CBZ, a state-dependent channel blocker that inhibits sodium current within seconds when applied externally, [18] is known to stabilize the inactivated current state of NaV1.3, NaV1.7, and NaV1.8 without targeting resurgent sodium current [19], [20]. Though the degree to which sodium channel blockade impacts TLR4 agonist-mediated effects is largely unknown, Black and colleagues have shown that phenytoin and tetrodotoxin substantially reduces phagocytic activity of microglia cells activated with the TLR4 agonist, lipopolysaccharide (LPS) [21]. Taken together, these TLR4-agonist-mediated events demonstrate that sodium channels may be possibly affiliated with TLR4 signaling as well as instrumental to the rapid induction of cellular activity in both immune cells and nociceptive sensory neurons.

The aim of this study was to assay the degree to which CBZ administration can diminish M3G/TLR4-induced neuronal activity *in vitro* and to characterize the potency of morphine, alone or in combination, with CBZ in the tibial nerve injury (TNI) model of neuropathic pain over time. We also determined the degree to which expression levels of NaV1.7 change following TNI paralleled changes in diminished morphine efficacy.

## Materials and Methods

Pathogen-free, adult female and male Sprague-Dawley (S/D) rats (150–200 g; Harlan Laboratories, Madison, WI) were housed in temperature (23±3°C) and light (12-h light: 12-h dark cycle; lights on at 07:00 h) controlled rooms with standard rodent chow and autoclaved tap water available. Experiments were performed during the light cycle. Animals were randomly assigned to the treatment groups. All animal experiments were approved by the Institutional Animal Care and Use Committees of Indiana University School of Medicine and College of Medicine, University of Arizona. All procedures were conducted in accordance with the Guide for Care and Use of Laboratory Animals published by the National Institutes of Health and the ethical guidelines established by the International Association for the Study of Pain.

### Tibial Nerve Injury

All rodents were anesthetized during the procedure with isoflurane (4% induction, 2% maintenance). To model neuropathic pain we performed a tibial nerve injury (TNI) as previously described [22], [23], [24], [25]. Rodents were anesthetized using isoflurane at 4% induction and 2% maintenance. Under anesthesia, the right sciatic nerve was isolated under aseptic surgical conditions by blunt dissection of the femoral biceps muscle, without damaging the epimycium. The sciatic nerve and its three branches were isolated: the sural, common peroneal and tibial nerves and only the tibial nerve was tightly-ligated with 5-0 silk and transected distal to the ligation. The removal of an additional 2–4 mm of distal nerve stump was removed to prevent re-innervation by the proximal nerve. The overlying muscle and skin was then sutured in two separate layers.

### Behavioral assessment

All rodents were habituated to testing chambers which were transparent plastic cages with a floor of wire mesh with ∼1 cm×1 cm openings for at least two days and baseline testing occurred for each of three days before TNI to establish force threshold necessary for paw withdrawal using previously described techniques [26], [27]. The incidence of foot withdrawal was expressed as a percentage of the 6 applications of each stimulus and the percentage of withdrawals was then plotted as a function of force. The von Frey withdrawal threshold was defined as the force that evoked a minimum detectable withdrawal observed on 50% of the tests given at the same force level. Following TNI, animal behavioral thresholds were assayed at least 30 minutes prior to drug administration and tested again following reagent administration.

### Drugs and Administration

Carbamazepine (CBZ) was purchased from Sigma (St. Louis, MO). Morphine sulfate salt and morphine-3-glucuronoide (M3G) was provided by NIDA Drug Supply Program (Rockville, MD). For *in vitro* studies, a 75 mM solution of CBZ was made up in dimethyl sulphoxide (DMSO). A 2.67-µl aliquot was taken directly from this stock and put into the 2 ml of bathing solution for the population data to obtain a final concentration of 100 µM [DMSO, 0.1% (v/v)]. For *in vivo* studies, CBZ was suspended in 5% DMSO (% of final volume) in saline (0.9%). Morphine was freshly prepared on the day of the experiment in saline such that the final dose was dissolved in 1 mL and administered by intraperitoneal (i.p.) injections.

### RNA isolation and RT-qPCR

Lumbar dorsal root ganglia (DRG; L4-L5) were dissected from adult Sprague Dawley rats, frozen in liquid nitrogen, and maintained at −80°C until processed for RNA extraction. Total RNA was extracted from the samples using the RNeasy RNA extraction and purification kit (Qiagen). Single stranded cDNA was synthesized using reverse transcriptase (Bioline) with oligo-dT primers. Quantitative PCR was performed as previously described [28]. Briefly, resultant cDNA samples were amplified on an ABI PRISM 7900 HT Sequence Detection System (Applied Biosystems) using the reporter, SYBR Green. The PCR reaction was as follows: 1x, 50°C, 2 min; 1x, 95°C, 10 min; 45x, 95°C, 15 s, 60°C, 1 min; 1x, 25°C, hold. To check for DNA contamination, PCR was run using an L27 (ribosomal housekeeping gene) primer pair, whose PCR product crosses an intron. The mRNA level for each gene (x) relative to L27 mRNA (internal control) was calculated using the following equation where Ct refers to threshold cycles: mRNA (x%) = 2^Ct(L27)-Ct(x)^×100.

Nav 1.7 For GAGAGCGGAGAGATGGATTC

Nav 1.7 Rev GCTTCAGTGGTTGTGATG

### Preparation of acutely dissociated dorsal root ganglion neurons

The L4–L6 DRGs were acutely dissociated using methods described by Ma and LaMotte [29]. Briefly, L4–L6 DRGs were removed from naive animals. The cells were then dissociated by mechanical trituration in culture media containing 1 mg/ml bovine serum albumin and trypsin inhibitor (Worthington Biochemical, Lakewood, NJ). The culture media was Ham's F-12 mixture, DMEM, supplemented with 10% fetal bovine serum, penicillin and streptomycin (100 µg/ml and 100 U/ml) and N2 (Life Technologies). The cells were then plated on coverslips coated with poly-L lysine and laminin (BD bioscience) and incubated for 2–3 h before more culture media was added to the wells. The cells were then allowed to sit undisturbed for 12–15 h to adhere at 37°C (with 5% CO2).

### Intracellular calcium imaging

Acute dissociation of lumbar DRG and ratiometric Fura 2-AM based intracellular calcium imaging was performed between 20–26°C using methods previously described [27]. Sterile solution was applied to cells prior to LPS followed by M3G application, and any cells that responded to buffer alone were not used. Compounds were applied directly into the coverslip bathing solution as a static solution. LPS (1 µg/mL) was applied first, followed by M3G (3 µM), after which capsaicin (3 nM; Sigma-Aldrich) was added (at least 100 cells were assayed from three rodents). Additional experiments utilizing at least 50 cells reversed the order of LPS and M3G. Only calcium imaging traces that reflected at least a 50% increase over baseline were included in the analysis. At least three minutes were allowed to pass between ligand inductions in order to allow neurons to return to a manifested baseline of calcium flux, to maintain the integrity of the results. If the neuron was unable to return to a baseline level, it was omitted from the study. All data were analyzed by two independent analyzers and only responses that were in agreement between the two individuals were used in the responsive cell counts.

### Electrophysiology

Sharp-electrode intracellular recordings were obtained from 4 to 18 hours after acute dissociation of lumbar DRG and current clamp protocols were performed using methods previously described [9], [22]. A neuron was accepted for study only when it exhibited a resting membrane potential (RMP) more negative than −45 mV. For each neuron isolated for study, a continuous recording was obtained for 1 minute without the delivery of any external stimulus. Neuronal excitability of small and medium diameter dissociated DRG sensory neurons was measured by injecting 1-s current pulses into the soma every 30 s. Current was adjusted in order to elicit 1–2 action potentials per current injection under baseline conditions. Following control current injections, M3G (3 µM) was applied to the coverslip and current injections continued every 30 s. Neuronal excitability was measured as number of action potentials elicited per current pulse before and immediately after addition of M3G (15 and 45 s, respectively). If M3G increased neuronal excitability, CBZ (2 mM) or vehicle was added to the bath to determine if M3G-elicited neuronal excitation could be reversed.

Whole cell voltage-clamp recordings were made from small- and medium-diameter DRG neurons (25 µm<DRG diameter <45 µm) using HEKA EPC10 amplifier (Germany). Electrodes were pulled from Warner Instruments thin walled borosilicate glass (Hamden, CT) with a Sutter P-97 electrode puller (Novato, CA) to produce 1–2 megaohm resistant pipets when filled with internal solutions. Recordings were performed in a reduced sodium solution to ensure fidelity of the voltage-clamp. External solution concentrations (in mM): 70 NaCl, 60 Choline-Cl, 30 TEA-Cl, 3 KCl, 1CaCl_2_, 1 MgCl_2_, 0.05 CdCl_2_, 10 HEPES, 10 Glucose (pH 7.3) (310–320 mOsm/L). Internal solution concentrations (in mM): 140 CsCl, 5 MgSO_4_, 10 EGTA, 4 ATP Na_2_-ATP, 25 HEPES (pH 7.2) (290–310 mOsm/L). Whole cell capacitance and series resistance were compensated by the amplifier, and linear leak currents were digitally subtracted by P/4. Analysis was performed using HEKA FitMaster (Germany) and Origin 9.1 (Northhampton, MA).

### Statistics

GraphPad Software (LaJolla, CA) was used to determine the statistical significance. Results were expressed as mean ± SEM. When only two groups were compared, Student t test was used. Multiple comparisons were evaluated by Tukey's multiple comparison tests after one-way ANOVA. p<0.05 was considered to be statistically significant.

## Results

### TLR4 agonist-induced intracellular calcium mobilization in dissociated sensory neurons

**Figure 1:**
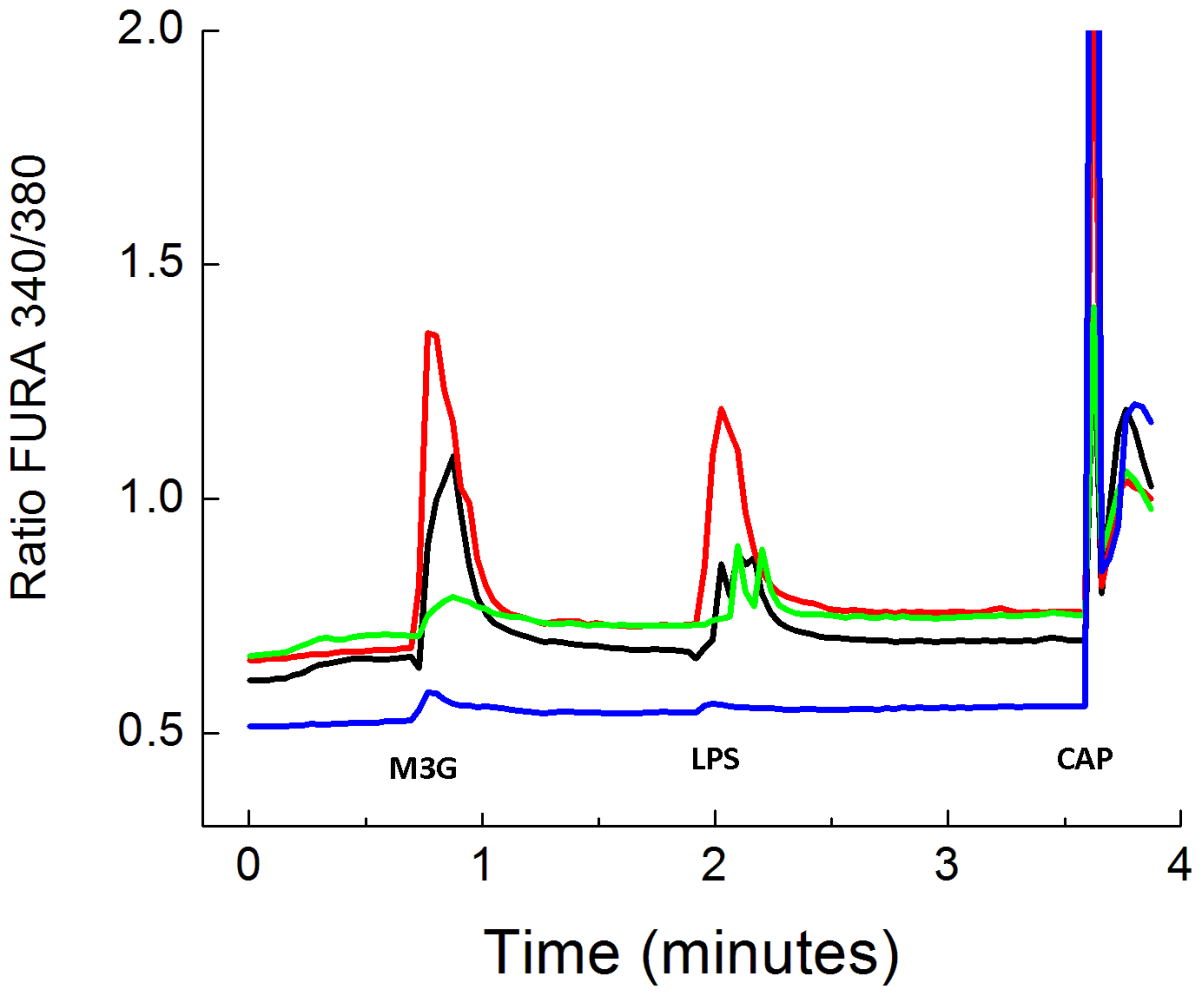
Description of calcium imaging. LPS-induced calcium flux in acutely dissociated small to medium diameter sensory neurons. Fura-2 loaded primary afferent neurons were stimulated with bath applications of morphine metabolite, morphine 3 glucuronide (M3G), the TLR4 agonist LPS, and capsaicin (CAP). [Ca2+]I levels were measured as ratio fluorscence of excitation at 340/380 nm over time. Four cells were assayed in this experiment and were represented by the blue, black green and red traces. The red and black traces clearly exhibited significant calcium flux after both M3G and LPS exposure while the green and blue traces exhibited only mild to moderate responses to these exogenous stimuli. All four traces exhibited robust calcium flux responses to capsaicin.

It is known that LPS can elicit excitation in sensory neurons in culture [9] and can contribute to the release of CGRP from capsaicin sensitive neurons [30]. Some reports suggest that this relatively fast neuronal activation may be dependent on both TLR4 and TRPV1 channels [8]. We therefore examined the effects of the known TLR4 agonists, LPS and M3G on [Ca^2+^]_i_ in cultured rat DRG neurons as a screen for the presence of functional receptors. Calcium mobilization was used in this instance strictly as an indication of TLR4 receptor activation and does not necessarily imply a role for this phenomenon in the other physiological effects of TLR4 agonists. The concentration of M3G and LPS used in this experiment was based on our previous observations of their effects on Ca mobilization in sensory neurons [9]. The application of vehicle at the beginning of experiment (i.e., 10 µl of 0.1% BSA/PBS in 1 ml of BSS) did not alter [Ca^2+^]_i_ (data not shown). Bath application of M3G followed by LPS exposure increased [Ca^2+^]_i_ in a large number of DRG neurons (**Fig 1** and **Table 1**). Many of these DRG neurons also responded to capsaicin (**Table 1**). These observations were maintained and supported regardless of whether the cells were first subjected to M3G followed by LPS, or vice versa.

**Table 1:** 

*Total Number of cells: 122*		
Treatment	Cell Number	Percentage
LPS responsive cells	(45/122)	36.89%
LPS/Capsaicin-sensitive cells	(21/45)	46.67%
M3G responsive cells	(19/122)	15.57%
M3G/LPS responsive cells	(18/19)	94.73%
M3G/LPS + Capsaicin-sensitive cells	(7/18)	38.88%

### Morphine-3-glucuronide-induced sensory neuron excitation *in vitro* is blocked by carbamazepine

**Figure 2:**
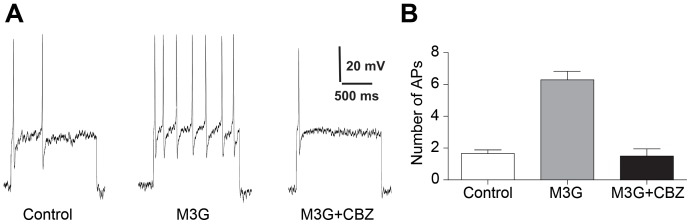
Morphine-3-glucuronide (M3G) increased excitability of nociceptive dorsal root ganglion neurons is reversed in the presence of carbamazepine (CBZ). (A) Current clamp recordings were performed on small (≥30 µm) to medium (≥40 µm) dorsal root ganglion (DRG) neurons (L1-6) from naïve rats. Firing of two to four action potentials (APs) was elicited by a 1 second depolarizing current injection (ranging from 0.1 to 0.6 nA depending on the cell) every 30 seconds. Representative recordings demonstrating that application of 3 µg/mL M3G increases the number of elicited APs. Bath applied CBZ reversed the effect of M3G-increased excitability. (B) Group data showing that M3G caused a significant increase in DRG AP firing that is reversed by CBZ (*P<0.05).

Activation of TLR4-mediated neuronal excitation by M3G selectively elicits increased density in the voltage-gated sodium currents NaV1.6, NaV1.7 and NaV1.9 [9] and CBZ is known to produce a differential block of NaV1.7 [20]. To investigate the degree to which M3G-induced neuronal excitation is affected by exposure to CBZ, we subjected sensory neurons to bath-applied M3G, followed by administration of CBZ. Not unlike our previous published observations [9], repeated current pulse combined with M3G administration produced a significant increase in the excitability of small- to medium-diameter sensory neurons when compared to baseline levels. Less than 18% of total neurons responded to M3G administration. We also observed 1.53±0.2 APs in cells under control conditions compared to 5.75±0.5 APs in cells subjected to M3G (n = 28) (Fig. 2A). Subsequent treatment with CBZ completely blocked M3G-dependent excitability in all sensory neurons that responded to M3G (1.13±0.3 APs for CBZ, n = 10; ANOVA, interaction F = 45.35, p<0.05; Dunnett's multiple comparison test, P<0.05) (Fig. 2B).

### Morphine-3-glucuronide-induced potentiation is dependent on voltage-gated sodium channels

**Figure 3:**
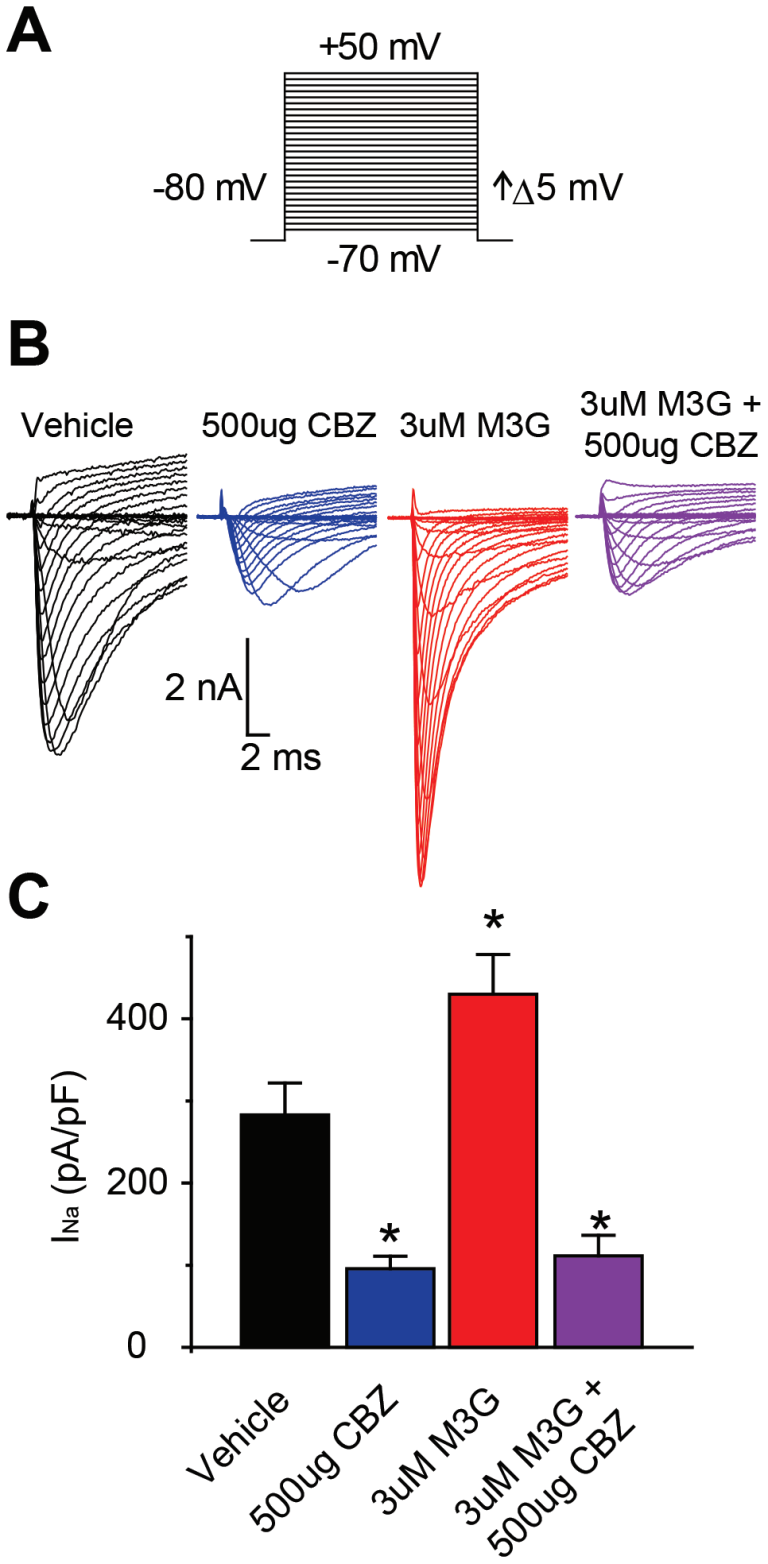
Morphine-3-glucuronide (M3G) potentiation of sodium currents is blocked by carbamazepine (CBZ). A, currents were elicited by performing voltage steps from a holding potential of −80 mV, in 5 mV increments, from −70 mV to +50 mV. B, representative traces from dorsal root ganglia cells treated with either 0.1% DMSO (Vehicle), 500 ug CBZ, 3 µM M3G, or both 3 µM M3G and 500 ug CBZ. C, peak currents (pA) were normalized to cell capacitance (pF) to measure current density (pA/pF). Asterisks indicate statistically significant differences between treatment groups and vehicle group (*P<0.05, one way ANOVA with Tukey's post-hoc test). Bars represent mean ± S.E.

Treatment with M3G potentiates currents in dorsal root ganglia cells [9]. CBZ has also been shown to inhibit voltage-gated sodium channel currents via hyperpolarizing shift in the voltage dependence of steady-state fast inactivation [20]. Here, we verify that the observed potentiation is dependent on voltage-gated sodium channels using whole-cell patch clamp technique and voltage step depolarizations from −80 mV to record isolated sodium currents and current densities from primary afferent sensory neurons (**Fig. 3A**). Sensory neurons were treated with an exposure to vehicle, 500 ug CBZ, 3 µM M3G, or a combination of both 3 µM M3G and 500 ug CBZ and current densities were compared between treatment groups (**Fig. 3B**; n = 24). CBZ treated cells measured 95.7±15.2 pA/pF, a ∼70% decrease in current density (**Fig. 3C**). M3G treated cells measured 430.0±48.3 pA/pF compared to 283.0±38.8 pA/pF for vehicle treated cells, a ∼50% increase in current density (**Fig. 3C**). Treatment with both M3G and CBZ measured 111.3±25.1 pA/pF, a ∼60% decrease in current density (**Fig. 3C**). Potentiation and reduction of current density by all treatment groups was measured to be statistically significant by one-way ANOVA and Tukey's post-hoc test. Thus, the potentiation of voltage gated sodium current mediated by M3G was attenuated by treatment with CBZ. Treatment with CBZ produced a characteristic hyperpolarizing shift in the voltage dependence of steady-state fast inactivation, −82.9±3.6 mV compared to −50.3±1.5 mV in vehicle treated cells (**Fig. 3C**). Treatment with both M3G and CBZ prevented this shift (**Table 2**).

**Table 2:** 

Condition	Activation		Fast Inactivation	
	V1/2	k	V1/2	k
Vehicle	−17.4±2.1 (n = 4)	6.7±0.7 (n = 4)	−50.3±1.5 (n = 4)	14.8±1.4 (n = 4)
500 ug CBZ	−12.4±1.7 (n = 5)	5.4±0.5 (n = 5)	−82.9±3.6 (n = 4)	17.7±2.7 (n = 4)
3 µM M3G	−17.0±2.1 (n = 5)	6.7±0.7 (n = 5)	−52.7±1.9 (n = 5)	18.5±2.3 (n = 5)
3 µM M3G + 500 ug CBZ	−21.4±0.4 (n = 3)	3.0±0.3 (n = 3)	−45.7±3.0 (n = 3)	11.1±2.1 (n = 3)

### Effect of Morphine on TNI-induced tactile allodynia at post-injury day 7–28

**Figure 4:**
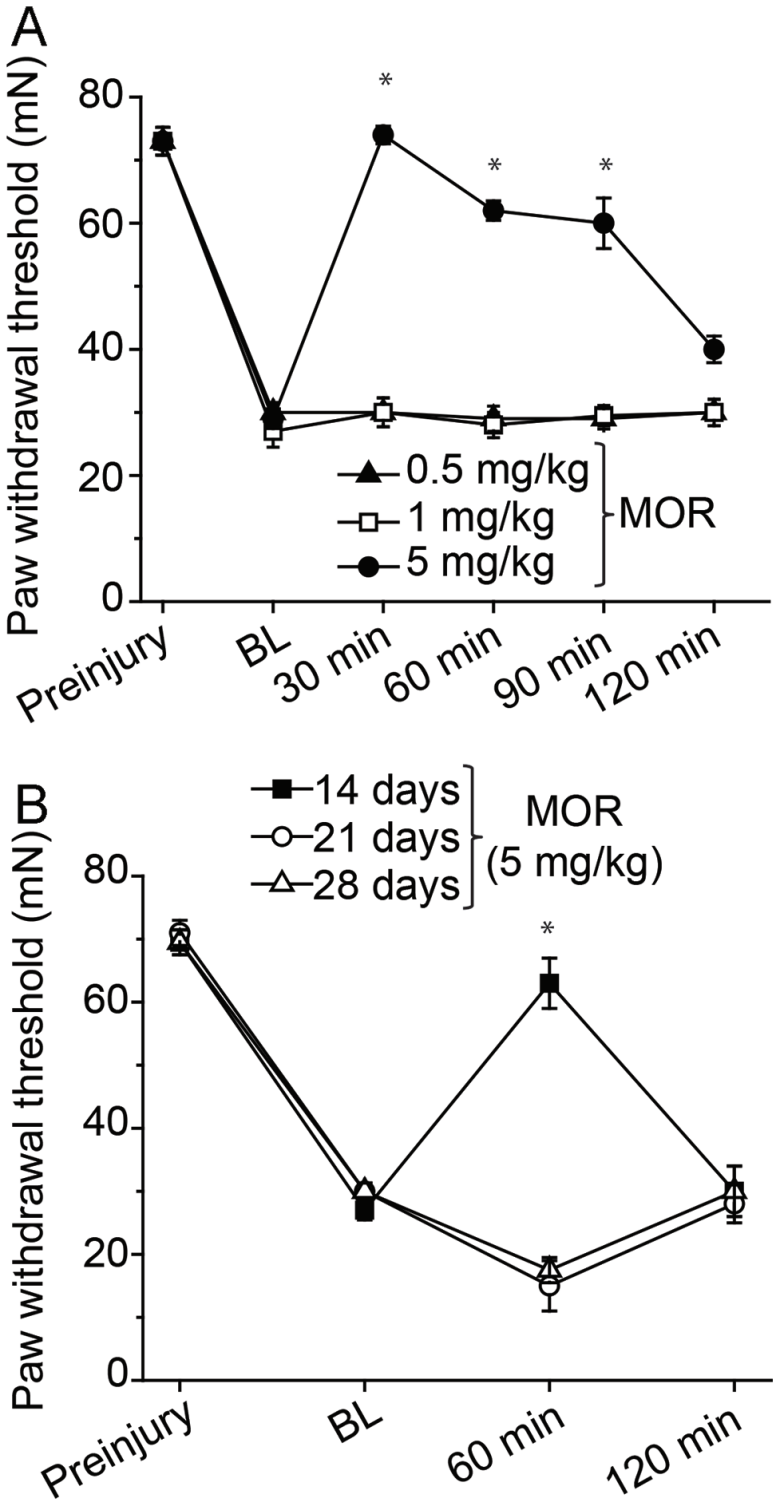
Dose- and time-effects of intraperitoneally administered morphine (MOR) on tactile allodynia in the tibial nerve injury (TNI) model at post-injury day 7. (A) The ability of MOR to attenuate tactile allodynia induced by TNI in a dose-dependent fashion was limited to 5 mg/kg. Each line represents the groups mean and SEM of 6–10 female rats. Drug group behavior at 30, 60, 90, or 120 minute vs TNI baseline (BL) behavior. (B) Time-dependent effects of intraperitoneally administered morphine (MOR) on tactile allodynia in the tibial nerve injury (TNI) model at post-injury day (PID) 14, 21 and 28. The ability of MOR to attenuate tactile allodynia induced by TNI was evident in PID 14 rats but not animals at PID21 or 28. Each line represents the groups mean and SEM of 6–10 rats. Drug group behavior at 60 minute or 120 minute vs TNI baseline (BL) behavior (*P<0.05).

Opioids such as morphine are the most effective broad spectrum analgesics available for moderate to severe pain acute pain conditions, however neuropathic pain tends to be less responsive to opioids. The ability of morphine to evoke pain relief diminishes over time and may be partially due to the ability of the opioid and the metabolite M3G to act through non-classic opioid receptors [31]. To identify the limits of morphine to reduce hyper-nociception following induction of a rodent neuropathic pain model, rats were subjected to a TNI procedure and tested at post-injury day 7 (PID7). All tested animals demonstrated signs of mechanical allodynia with ipsilateral threshold values of 28.7±2.5 mN compared with pre-surgery withdrawal threshold values of 72.1±5.0 mN (n = 64; p<0.001) (**Fig. 4A**). The injured animals continued to exhibit mechanical allodynia for as long as they were monitored. Body weight and general welfare (grooming/socialization) of the animals were routinely observed and appeared to be unaffected by the nerve injury.

The dose-response function for the morphine reversal of TNI pain at PID7 was determined using a within-subjects design. Rats were habituated and baseline testing conducted as described in the methods. Not unlike previous work by Erichsen and Blackburn-Monro [32], systemic administration of low dose morphine (0.5 or 1 mg/kg, i.p.) had no effect on withdrawal threshold up to 120 min post-injection for the ipsilateral hindlimb compared with either the pre-injection withdrawal threshold or with vehicle-treated animals at the corresponding time points. In contrast, i.p. injection of morphine at 5 mg/kg significantly increased withdrawal threshold of the ipsilateral hindlimb 30 min after injection to 73±1.6 mN (F = 12.81, p<0.05), compared with baseline and vehicle), and this effect continued up to 90 min, before returning to a level of sensitivity comparable with baseline and vehicle at 120 min (**Fig. 4A**).

To test the degree to which the 5 mg/kg (i.p.) concentration of morphine is effective for the reversal of TNI-induced tactile allodynia across time, independent groups of injured rats at PID 14 (n = 8), 21 (n = 8), and 28 (n = 8) were also assayed for mechanical allodynia following morphine administration. At a dosage of 5 mg/kg of morphine in the rodents, tactile allodynia was reversed in PID 14 animals. However, this dosage proved ineffective at PID 21 or 28, with no significant differences in pain behavior (F = 48.14, p<0.05, **Fig. 4B**).

### Combination of morphine and carbamazepine reverses TNI-induced tactile allodynia at post-injury day 28 in a dose dependent manner

**Figure 5:**
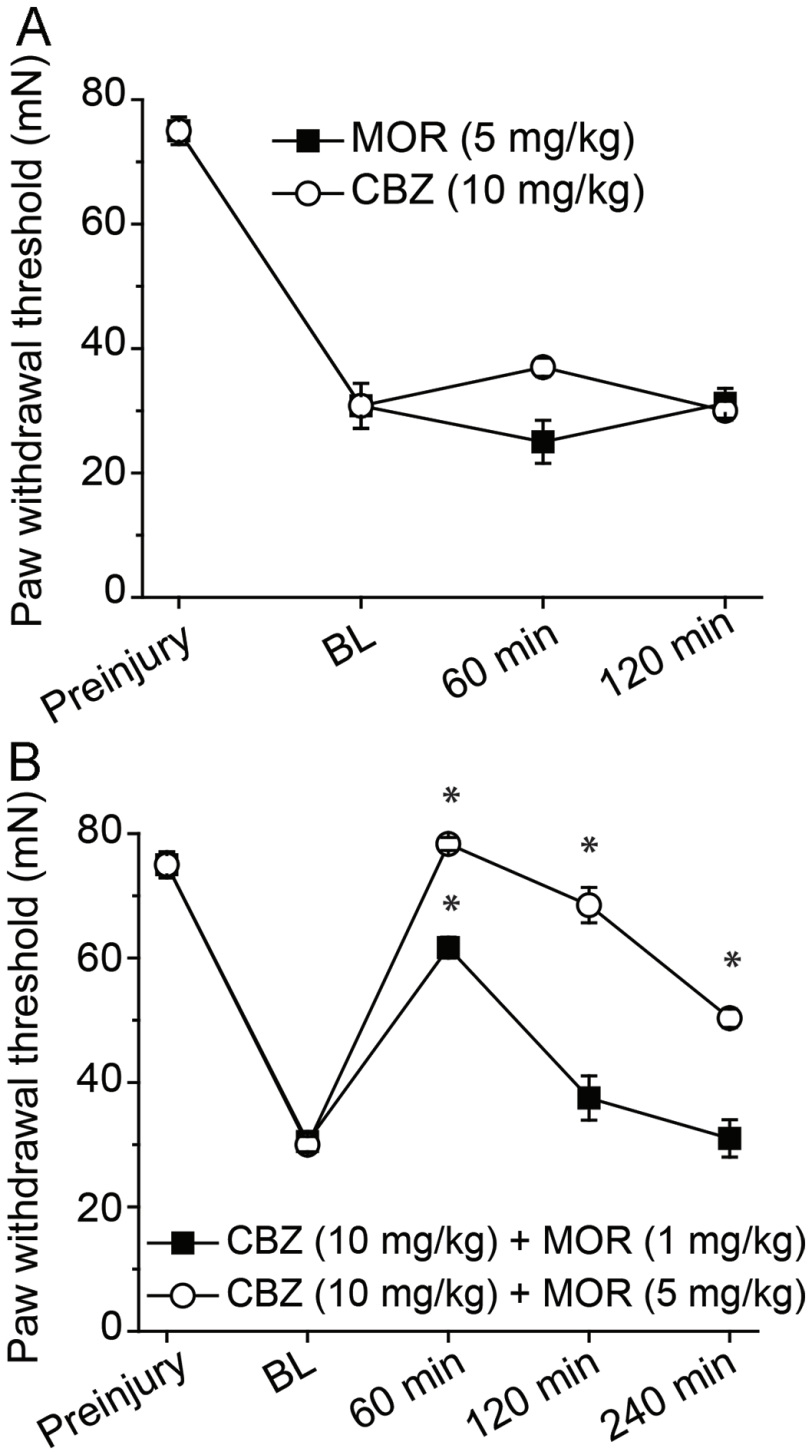
The effect of intraperitoneally administered morphine (MOR), carbamazepine (CBZ) or combination of MOR/CBZ on tactile allodynia in the tibial nerve injury (TNI) model at post-injury day (PID) 28. (A) Neither MOR or CBZ affect tactile allodynia induced by TNI. Each line represents the groups mean and SEM of 6–10 female rats. Drug group behavior at 60 minute or 120 minute vs TNI baseline (BL) behavior. (B) Effect of co-administered MOR and CBZ on tactile allodynia in the tibial nerve injury (TNI) model at post-injury day 28. The ability of MOR/CBZ to attenuate tactile allodynia induced by TNI was dose-dependent. Each line represents the groups mean and SEM of 6–10 rats. (*P<0.05; combination therapy group vs TNI baseline (BL) behavior).

It is known that administration of M3G can induce tactile allodynia through a TLR4-mediated mechanism in the absence of nerve injury [9]. That neuronal exposure to M3G and the associated potentiation of NaV currents in vitro can be reversed by CBZ suggests a potential benefit of a combination pharmacotherapy approach using morphine and CBZ *in vivo* for TNI-induced tactile allodynia. To first determine whether CBZ alone is effective for TNI-induced tactile allodynia, we administered CBZ 10 mg/kg i.p. to TNI rodents at PID28 and compared the outcomes to similar injections of morphine 5 mg/kg i.p. at PID 28. Neither drug negated the injury-induced behavior (F = 19.2, p<0.05, **Fig. 5A**).

The potential impact of NaV1.7 in the injured peripheral nervous system may be pain syndrome specific as determined by NaV1.7 knockout mouse strains [33] and gain of NaV1.7 function appears to result in congenital insensitivity to pain or lead to severe familial pain disorders [34]. However diminishing the off-target effects of morphine directly or via its metabolite M3G may offer increased analgesic value for the widely used clinical opioid. As a proof-of-concept, morphine was combined with CBZ to produce a novel combination pharmacotherapy. In the 2-drug-combination studies, morphine plus CBZ completely attenuated mechanical allodynia at 5 mg/kg and 1 mg/kg concentration (F = 19.2, p<0.05, **Fig. 5B**). Additional analysis revealed that there was increased efficacy with the combination pharmacotherapy of 5 mg/kg morphine and 10 mg/kg CBZ as PWTs following administration of both drugs did not return to pre-injury levels for at least 240 minutes in the PID28 rodent group. Similar observations were present in age-matched male rodents subjected to TNI (data not shown; n = 6–8 per post injury time point).

### Increased NaV1.7 expression following tibial nerve injury by post-injury day 28

**Figure 6:**
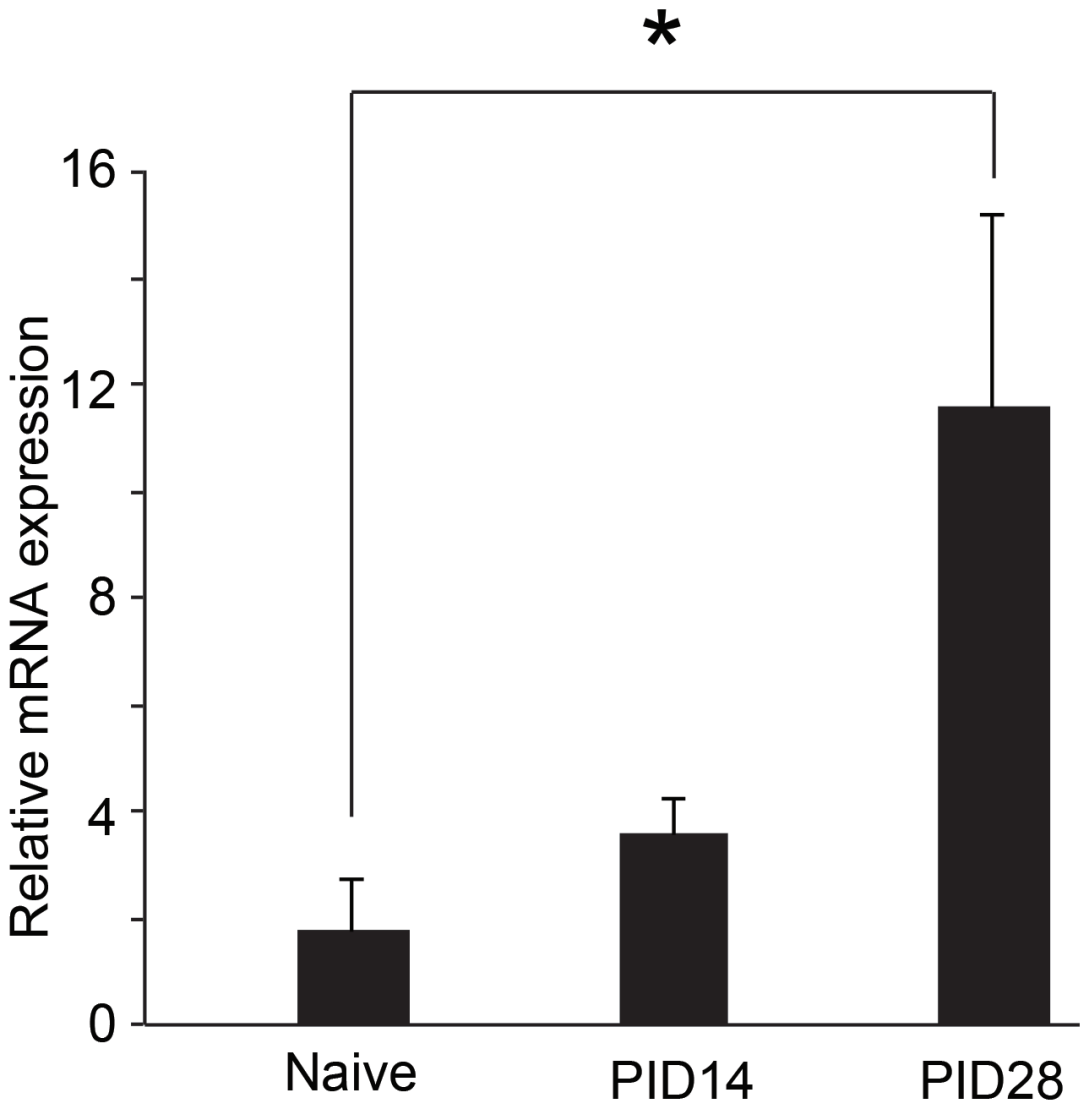
Tibal Nerve Injury (TNI) alters the expression of neuronal transcripts in rat dorsal root ganglion (DRG). RT-PCR analysis showing the mRNA expression profile of NaV1.7 in the DRG derived from naïve animal and at different time points following TNI; post injury day (PID) 14 and PID 28 (n = 3). RT-PCR data were analyzed using the Ct method and mRNA expression levels are expressed relative to L27- ribosomal housekeeping gene. (*P<0.05; TNI gene expression vs naive threshold baseline).

Given the relative selectivity of CBZ for the NaV1.7 current state [20] and a recent report by Laedermann and colleagues which describes spared nerve injury-induced increases in NaV1.7 [35], [36], we sought to determine the degree to which TNI changes in NaV1.7 correspond with morphine efficacy across time. Based on a quantitative real time PCR analysis, it appears that rodents subjected to TNI exhibited substantial increases in NaV1.7 mRNA by day 28 compared to naïve tissue levels, whereas post-injury day 14 values did not reach statistical significance (**Fig. 6**; n = 4, ANOVA, F = 5.571; p<0.05).

## Discussion

It is known that opioids such as morphine and opioid metabolites induce glial-associated inflammatory responses within the brain and spinal cord via the non-classical opioid receptor, TLR4 [12]. Ligand activation of neuronal TLR4 has also been observed in the nervous system [8], [10], [37] and may play a direct role in the diminished efficacy of opioids in neuropathic pain conditions. The present set of multidisciplinary studies provide converging lines of evidence that TLR4-mediated increased neuronal excitation is likely dependent upon the CBZ-sensitive sodium current, and that the latent increase in NaV1.7 mRNA expression in sensory ganglia following TNI contributes to decreased opioid efficacy in a rodent model of neuropathic pain.

The use of opioids such as morphine to treat neuropathic pain is controversial due to the mixed results of clinical studies and is considered to be a second or third tier of treatment at best [38] [39]. Pre-clinical evidence indeed supports the observation that the analgesic effects of morphine are greatly diminished in the setting of neuropathic pain and quite often necessitates greater concentrations of morphine to provide pain relief. The mechanisms associated with these changes in opioid efficacy are often attributed to opioid tolerance. However, clear examples of the loss of morphine analgesia are evident in models of neuropathic pain such as spared nerve injury. Erichsen and Blackburn-Monro first demonstrated that this type of injury model required systemic doses of up to 6 mg/kg morphine for nociceptive pain relief at day 7 [32]. In contrast, doses of morphine as high as 10–30 mg/kg are necessary to provide relief for neuropathic pain at post-injury day 14 [40], [41]. A comparison of the opioid dose needed to reverse mechanical allodynia in the present study appear to parallel these previous observations as the efficacy of the morphine dose that provided opioid analgesia up to day 14 was completely lacking at injury day 21 or 28. Together, these data readily indicate that the efficacy of morphine alone in attenuation of tactile allodynia is refractory over time following injury-induced changes in the neurobiology of the rodent.

Though the analgesic mechanism of opioids is largely thought to modulate transmission of pain sensation at the level of the spinal cord or by altering the perception of painful stimulus/stimulus-independent pain at cortical regions [42], it is conceivable that cell membrane bound receptors other than opioid receptors in either the spinal cord or the peripheral nervous system may contribute to the diminished efficacy of opioids in neuropathic pain conditions over time. For example, numerous lines of evidence suggest that the pathogenesis of neuropathic pain is dependent in part on abnormal spontaneous activity within sensory neurons [29], [43], [44]. As this spontaneous activity is likely dependent on NaVs for the generation and conduction of action potentials, it is conceivable that neuronal hyperexcitability after injury is dependent on injury-induced changes in specific isoforms of NaV channels including NaV1.7 [45]. Molecular mechanisms which may contribute to the latent changes in sodium channel expression include the ubiquitin protein ligase known as neuronal precursor cell expressed developmentally downregulated-4 type 2 or NEDD4-2 [35]. NEDD4-2 was recently identified as a posttranslational regulator of a number of different sodium channels including NaV1.7 [35]. Subsequently reduced levels of NEDD4-2 due to peripheral nerve injury may contribute to the development of both neuropathic pain disorders and diminished analgesic efficacy of opioids.

Expression of both Nav1.7 and TLR4 may endow small nociceptive neurons with rapid repriming abilities that contribute to spontaneous discharge or hyperexcitability in the presence of opioids or opioid metabolites. To potentially control for the off-target effect of morphine or its metabolite M3G, we utilized CBZ in combination with morphine. CBZ is known to produce a substantial block of electrical activity associated with NaV current in sensory neurons and stably transfected human embryonic kidney 293 cells expressing wild-type NaV1.7 independent of resurgent currents [19], [20]. Given the apparent effects of CBZ blockade of NaV current, it is reasonable to conclude that the combination of morphine with CBZ diminishes opioid-induced enhancement of electrical activity potentially associated with NaV1.7 in nociceptive sensory neurons while the central nervous system effects of morphine elicits analgesia sufficient to influence nociceptive behavior in the TNI model of neuropathic pain. However, evidence contained herein also suggests that responsive neurons elicit a range of current changes following exposure to M3G, all of which are greater that the averaged control value. This range of effects may be dependent on varying extents of expression of necessary pathway proteins in the heterogeneous system [9], [46]. Although not tested in this study, it is unlikely that other anti-epileptic drugs such as gabapentin and pregabalin elicit similar effects as neither compound directly affects sodium current in a similar fashion [47].

The neuronal excitability mediated by opioids or their metabolites via TLR4 could also be influenced by signaling downstream of the receptor and may influence both capsaicin and non-capsaicin sensitive neurons. For example, it is possible that coordinated interactions between TLR4 ligands and sensory neurons could evoke TNFα-dependent tactile hypersensitivity and may be indicative of signaling dependent on the MyD88 signaling pathway [48]. Other proinflammatory cytokines which could conceivably be altered by the neuronal TLR4/MyD88 pathway including interleukin-1β [49] and deserves further investigation. However, the observed M3G-related neuronal signaling events are rapid and may be independent of signal transcription factors. Alternatively, the assembly of TLR adapter signaling proteins and activation of phospholipids in neurons may only require activation of a signal-transduction pathway leading to the observed increase in NaV current [50]. Additional studies are also underway to determine the degree to which hormonal regulation of neuronal TLR4 responses serve to influence neuropathic pain states alone or in combination with opioids [48], [51].

The apparent lack of morphine efficacy *in vivo* may depend on the increased expression of NaV1.7 in the rat DRG following injury to the peripheral nerve. Given the negligible change of gene expression change up to PID14, morphine administration serves as an effective means by which to transiently reverse TNI-induced mechanical allodynia. However, the observed pronounced increase in NaV1.7 expression observed by PID28 in the rat supports the functional change in the inability of morphine to attenuate mechanical allodynia. The degree to which injury produces similar effects in murine spared nerve injury models is unknown and may vary from species to species [36].

It is likely that abnormal functioning of sodium channels in the peripheral nervous system is a key event in both the etiology of neuropathic pain [52] and opioid-induced hyperalgesia [9]. Subsequently, there are potential avenues for the use of morphine in combination with several FDA-approved partial seizure therapeutics currently known to affect the NaV1.7 current; lacosamide [20] or rufinamide [53] or possibly the anti-depressant duloxetine [54]. Oxcarbazepine, as a structural derivative of CBZ, may also affect NaV1.7 and serve as a suitable combinatorial therapeutic in combination with morphine for chronic pain (see Clinical Trials Registry No. NCT02078089). In contrast, anti-convulsive therapeutics that target focal seizure disorders by acting on voltage-sensitive calcium channels may not be able to diminish the off-target effects of opioids or provide enhanced opioid efficacy [55].

Together, the increased neuronal excitability via opioid-metabolite mediated neuronal TLR4 signaling increased NaV current in sensory neurons may serve as a robust signal to dampen the analgesic effects of morphine over time. However, here we show that the dampening of the analgesic effects of morphine on neuropathic pain behavior in vivo can be countered with the addition of CBZ. Thus, the same peripheral sensory neurons may possess opposing properties that elicit a critical interplay of mechanisms which may be central to the understanding of tolerance and paradoxical allodynia that can occur with opioid-based therapies.
